# Perfect energy stability in neurons

**DOI:** 10.18632/aging.102257

**Published:** 2019-09-09

**Authors:** L. Felipe Barros, Felipe Baeza-Lehnert

**Affiliations:** 1 Centro de Estudios Científicos - CECs, Valdivia, Chile

**Keywords:** homeostasis, ATP/ADP, Na+/K+ ATPase pum, glycolysis, oxidative phosphorylation

Neurons invest considerable energy in the generation and maintenance of a steep electrochemical Na^+^ gradient across the plasma membrane. The Na^+^ gradient is in turn used for various housekeeping tasks, including import of nutrients, export of waste, volume and pH regulation, and modulation of Ca^2+^signaling. In addition, neurons make intensive use of the Na^+^ gradient to transmit information by membrane depolarization, to the extent that in these cells the Na^+^/K^+^ ATPase pump accounts for over 80% of the ATP turnover. Almost all ATP is produced in mitochondria, so how does the organelle know how much to produce and when? This is a central question in bioenergetics. Ever since the studies of isolated mitochondria by Britton Chance and colleagues in the hay days of cybernetics and feedback control theory, the coupling between energy demand and supply has been assigned to ADP [1]. Later on Krebs cycle dehydrogenases and other mitochondrial enzymes were found to be sensitive to Ca^2+^ [2,3], suggesting an additional coupling mechanism.

Now is the era of genetically-encoded fluorescent sensors, which afford improved spatial and temporal resolution for the study of metabolism in intact cells [4]. Equipped with these tools, our group set out to map the first seconds of the neuronal response to workload [5]. The initial results were ostensibly boring. Neuronal ATP, ATP/ADP, glucose, pyruvate, lactate and pH were all found to be unaffected by excitatory neurotransmission. However, flux measurements using transporter-stop protocols unveiled a rich underlying phenomenology. Neuronal ATP consumption had been stimulated by 200%, while glycolytic flux and mitochondrial pyruvate consumption had increased by 200-300%. The stability was therefore explained not by lack of demand but by a close second-to-second match between ATP consumption and production. *In vivo* determinations in the somatosensory cortex confirmed the stability of neuronal ATP through bouts of activity. How could ADP or ATP possibly play any regulatory role if their concentration does not change? Next we turned to the alternative hypothesis but found the cytosolic Ca^2+^ had already returned to resting level at the time of maximum ATP turnover, and that intramitochondrial Ca^2+^ did not even rise. Further experiments indicated that the Na^+^ pump itself controls mitochondria, acting via an unspecified mechanism that may involve the glycolytic machinery [5].

The inordinate metabolic stability of neurons is exciting. Is neuronal ATP depletion a reflection of high level physiological stimulation or is it frankly pathological? In support of the latter, both tetanic stimulation and glutamate impaired mitochondrial pyruvate consumption, showing that the ATP depletion is partly secondary to a drop in production [5]. ATP depletion affects scores of reactions and under certain conditions may trigger a runaway metabolic collapse [6]. Neurons must endure a lifetime of unpredictable activations without the benefit of energy stores. Could repetitive cycles of ATP depletion over years of dysfunctional adaptation contribute to neurodegeneration? The machinery that links the Na^+^ pump to mitochondria to ensure perfect energetic stability in neurons may hold clues to these important questions.
